# Genetics of Hepatocellular Carcinoma: From Tumor to Circulating DNA

**DOI:** 10.3390/cancers15030817

**Published:** 2023-01-28

**Authors:** Claudia Campani, Jessica Zucman-Rossi, Jean-Charles Nault

**Affiliations:** 1 Centre de Recherche des Cordeliers, Sorbonne Université, Inserm, Université de Paris Cité, Team «Functional Genomics of Solid Tumors», 75006 Paris, France; 2 Equipe labellisée Ligue Nationale Contre le Cancer, Labex OncoImmunology, 75006 Paris, France; 3 Internal Medicine and Hepatology Unit, Department of Experimental and Clinical Medicine, University of Firenze, 50134 Firenze, Italy; 4 Hôpital Européen Georges Pompidou, APHP, 75015 Paris, France; 5 Liver Unit, Hôpital Avicenne, Hôpitaux Universitaires Paris-Seine-Saint-Denis, Assistance-Publique Hôpitaux de Paris, 93000 Bobigny, France; 6 Unité de Formation et de Recherche Santé Médecine et Biologie Humaine, Université Paris Nord, 93000 Bobigny, France

**Keywords:** hepatocellular carcinoma, genomics, target therapies, biomarkers, liquid biopsy, circulating tumor DNA

## Abstract

**Simple Summary:**

In recent years, the genetic landscape of hepatocellular carcinoma (HCC) has been explored, identifying *TERT* promoter, *CTNNB1*, and *TP53* as the most frequent mutated genes. Therapies directed against specific targetable genomic alterations are the basis of personalized medicine and represent the cornerstone of systemic treatment for many malignancies, but are not yet available in HCC. Tools such as liquid biopsy and, in particular, circulating tumor DNA (ctDNA) may help in identifying biomarkers of response or resistance to treatment, and their role in HCC is an active field of research. In this review, we summarize the available evidence on the HCC genomic landscape and the potential role of ctDNA in clinical practice.

**Abstract:**

Hepatocellular carcinoma (HCC) accounts for 90% of primary hepatic malignancies and is one of the major causes of cancer-related death. Over the last 15 years, the molecular landscape of HCC has been deciphered, with the identification of the main driver genes of liver carcinogenesis that belong to six major biological pathways, such as telomere maintenance, Wnt/b-catenin, P53/cell cycle regulation, oxidative stress, epigenetic modifiers, AKT/mTOR and MAP kinase. The combination of genetic and transcriptomic data composed various HCC subclasses strongly related to risk factors, pathological features and prognosis. However, translation into clinical practice is not achieved, mainly because the most frequently mutated genes are undruggable. Moreover, the results derived from the analysis of a single tissue sample may not adequately catch the intra- and intertumor heterogeneity. The analysis of circulating tumor DNA (ctDNA) is broadly developed in other types of cancer for early diagnosis, prognosis and monitoring under systemic treatment in order to identify primary and secondary mechanisms of resistance. The aim of this review is to describe recent data about the HCC molecular landscape and to discuss how ctDNA could be used in the future for HCC detection and management.

## 1. Introduction

Hepatocellular carcinoma (HCC) is the most common primary liver cancer, accounting for approximately 90% of cases and representing the third leading cause of cancer related deaths worldwide [1]. In 80 to 90% of the cases, HCC develops on a background of cirrhosis due to hepatitis B virus (HBV) and hepatitis C virus (HCV) infection, chronic alcohol consumption and non-alcoholic fatty liver disease (NAFLD) [1].

Interestingly, some single nucleotide polymorphisms (SNPs), inherited genetic variants observed in more than 5% of the population that could influence the occurrence and clinical phenotype of human diseases, have also been associated with HCC development, mostly in alcoholic liver disease and non-alcoholic liver disease. Patatin-like phospholipase domain-containing protein 3 (*PNPLA3)* rs738409 C>G (p.I148M) [2,3,4,5,6] and transmembrane 6, superfamily member 2 (*TM6SF2)* rs58542926 C>T [7,8,9] have been associated with non-alcoholic steatohepatitis (NASH) and alcohol-related HCC and membrane-bound O-acyltransferase domain containing 7 (*MBOAT7)* rs641738 C>T has been associated with NAFLD-related HCC [10]. Conversely, the 17β-Hydroxysteroid dehydrogenase 13 (*HSD17B13)* rs72613567 and Wnt Family Member 3A and 9A (*WNT3A-WNT9A)* rs708113 variants have been identified to be protective of HCC development in patients with alcoholic liver disease [11,12,13]. 

The prognosis of HCC is still poor, with a 5-year survival of approximately 18% as the majority of patients are diagnosed at stages where curative therapies are not possible [14]. For this reason, tools that enable an early diagnosis with a greater sensitivity than those currently used (ultrasonography with or without alpha-foetoprotein (AFP)) [15,16,17] are urgently needed. Moreover, the field of systemic treatment of HCC has rapidly evolved and currently six different first- and second-line treatments are approved [15,18]. In light of the increasing number of systemic therapies available [19], the development of biomarkers that enable the identification of patients who are more likely to respond to a specific treatment or that identify acquired resistance occurrence is still an unmet need. Although several biomarkers have been studied with these purposes, none are currently validated for early HCC detection, prognosis assessment, and prediction of response to systemic therapies, with the exception of serum AFP, that helps to select patients who will benefit from ramucirumab [20].

In this review, we aim to describe the major recent advances in genomic studies of HCC and to explore the potential role of circulating tumor DNA (ctDNA) as a biomarker in clinical practice. 

## 2. Mutational Landscape of Hepatocellular Carcinoma

**Figure 1:**
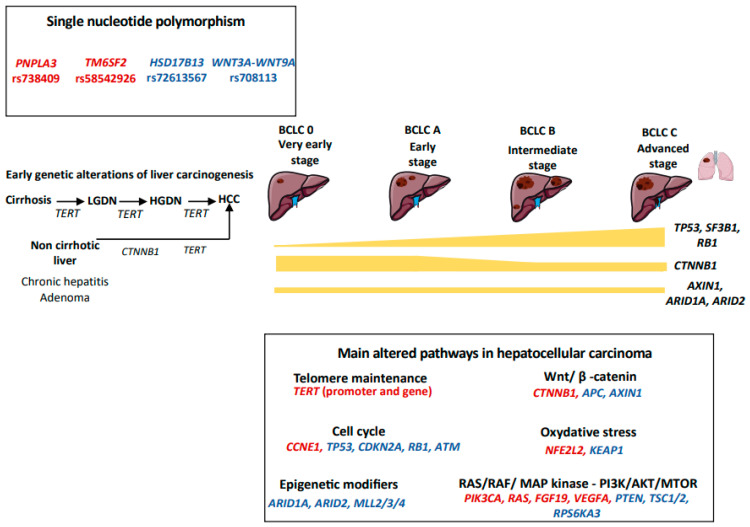
Genetic landscape of hepatocellular carcinoma. We figured the main somatic genetic drivers observed in hepatocellular carcinoma, their frequencies across BCLC stages and the main signaling pathways dysregulated. Activating mutations are reported in red, whereas inactivating mutations are in blue. LGDN: low-grade dysplastic nodule; HGDN: high-grade dysplastic nodule; HCC: hepatocellular carcinoma.

HCC results from the accumulation of genomic and epigenomic alterations in hepatocytes and its development is shaped by the tumor microenvironment. Next Generation Sequencing (NGS) techniques have increased our knowledge of the genetic diversity of HCC [21,22,23]. HCC is characterized by a tumor mutational burden of two to nine mutations per megabase that corresponds to approximately 50–70 somatic alterations in protein-coding regions which accumulate during the lifetime of hepatocytes [21,24]. However, the majority of these mutations are “passengers” and only two to six of them occur in “driver” genes that promote liver carcinogenesis [21,24]. The main driver genes involved in liver carcinogenesis impact six key biological pathways: telomere maintenance, Wnt/ β-catenin, cell cycle regulation, epigenetic dysregulation, oxidative stress and activation of RAS/RAF/ MAP kinase and PI3K/AKT/MTOR pathways (summarized in Figure 1). To note, HBV also has peculiar ways to drive liver carcinogenesis, including the action of a viral oncoprotein as well as the integration of the virus in the human genome. HBV insertional mutagenesis viral enhancer nearby cancer-driver genes may lead to the overexpression of oncogenes, but may also trigger chromosome rearrangements leading to gene alterations at a distance [25].

### 2.1. Telomerase Maintenance

The most frequently altered pathway in HCC is linked to telomerase reactivation [26]. Telomerase is a complex composed of telomerase reverse transcriptase (TERT), telomerase RNA component (TERC) and various other proteins (e.g., TRF1, TRF2, TIN2, RAP1, TPP1, and POT1) that enables telomere synthesis [27]. This complex is physiologically suppressed in most cells of the adult body, including mature hepatocytes [28]. Preventing telomere shortening after cell divisions, telomerase reactivation favors cell immortalization and the accumulation of genomic alterations that lead to carcinogenesis [29]. We have previously shown than aging, liver fibrosis, male sex and excessive alcohol consumption were the determinants of liver telomere attrition [30]. Particularly, cirrhosis was characterized by the presence of short telomeres, underlying the need to reactivate telomerase in order to promote malignant transformation and proliferation [30]. Different mechanisms of telomerase reactivation have been described in HCC: *TERT* promoter mutations, viral insertion (HBV or adeno-associated virus type 2 (AAV2)) in the *TERT* promoter, *TERT* amplification and translocation [29]. In addition, the alternative lengthening of telomeres mechanism has been described in a small subset of tumors [29,31]. Somatic *TERT* promoter mutations are the most frequent somatic mutations found in HCC (30-60%), and in 95% of cases are represented by a C>T transition in the −124bp hotspot [29] and co-occur frequently with mutations in catenin beta 1 (*CTNNB1*) [21]. To note, *TERT* promoter hotspot mutations are equally distributed across all Barcelona Clinic Liver Classification (BCLC) stages (from 50% in BCLC-0 to 60.6% in BCLC-C stages) [32] and could be also found in low-grade and high-grade dysplasia nodules (6% and 19%, respectively), highlighting the role of *TERT* alterations in the earliest steps of liver carcinogenesis [33,34]. While *TERT* hotspot mutations are frequently found in patients with chronic alcohol disorder and HCV-related HCC, in HBV-patients, telomerase is usually reactivated through the viral insertion in the *TERT* promoter, a genomic alteration found in approximately 26% of HBV-related HCC [29,35,36]. These data explain the heterogeneous geographical distribution of *TERT* promoter hotspot mutations, usually more frequent in Western countries than in Asia, where the HBV etiology is predominant [37]. A similar mechanism of viral genome integration in the *TERT* promoter has been also described for AAV2 [38], whose genome contains viral enhancers and transcription factor binding sites that favor *TERT* overexpression [38]. Less frequently (5% of cases), telomerase reactivation is linked to *TERT* amplification at DNA level or the fusion of *TERT* with highly expressed genes (e.g., Solute Carrier Family 12 Member 7 (*SLC12A7*) and Solute Carrier Family 7 Member 2 (*SLC7A2*)) [29].

### 2.2. Wnt/β-Catenin Pathway

The Wnt/β-catenin pathway is dysregulated in a large number of cancers, including HCC [26]. In approximately 37% of cases, the activation of the Wnt/β-catenin pathway in HCC is secondary to activating mutations of *CTNNB1*. Most of the mutations occur in a hotspot situated in the exon 3 at serine/threonine sites, or neighboring amino acids, altering the domain targeted by the adenomatous polyposis coli (APC)/AXIN1/ glycogen synthase kinase 3 (GSK3B) inhibitory complex. *CTNNB1* mutations protect β-catenin from degradation and lead to its accumulation at the nuclear level, where β-catenin activates target genes of the pathway, leading to aberrant proliferation of the tumor cell [26]. *CTNNB1* mutations are more frequent in patients with chronic alcohol consumption than in HCC related to other etiologies [32]. In addition, *CTNNB1* mutations are almost never identified in dysplastic nodules, suggesting that these alterations are not involved in the initiation of carcinogenesis in cirrhotic patients [39]. Conversely, mutations in exon 3 of the *CTNNB1* gene are observed in 10–15% of hepatocellular adenoma and are associated with an increased risk of malignant transformation into HCC, with the acquisition of the *TERT* promoter mutation as a second hit [40]. Wnt/β-catenin pathway activation is not only related to *CTNNB1* mutations, but could be induced by inactivating mutations of axin 1 (*AXIN1*) or *APC* in 15% and 2% of HCC cases, respectively [26]. Interestingly, *CTNNB1* and *AXIN1* mutations are mutually exclusive [21]. Moreover, even if *AXIN1* has been generally described as a negative regulator of the Wnt/β-catenin pathway, some studies reported that *AXIN1*-mutated HCCs also harbored dysregulation of the Notch and Yes-associated protein 1 (YAP) pathways [41]. 

### 2.3. Cell Cycle Regulation

Mutations of tumor protein 53 (*TP53*) are observed in approximately 20–50% of HCC [26]. Most *TP53* mutations change single amino acids in p53, leading to the production of an altered version of the protein, leading to an uncontrolled cell proliferation and resistance to apoptosis when DNA are exposed to mutations [42]. *TP53* mutations are more frequent in HBV-related HCC, and the G > T transversion at codon 249 of *TP53* (R249S) represents the molecular hallmark of aflatoxin B1 exposure [43]. In contrast to *TERT* promoter mutations, *TP53* alterations are not usually found in preneoplastic lesions [39] and their incidence increased progressively from early to advanced HCC (15.5% BCLC-0 vs. 35% BCLC-C) [32]. While a positive epistatic interaction has been described for *TP53* mutations and Kelch-like ECH-associated protein 1 (*KEAP1*), Tuberous Sclerosis Complex 2 (*TSC2*) mutations and Cyclin D1 (*CCND1)/* Fibroblast growth factor (*FGF19*) amplification, *TP53* mutations and *CTNNB1* are mutually exclusive [21]. Although less frequent, the retinoblastoma (RB) pathway, which controls the progression from the G1 to the S phase of the cell cycle, is also involved in liver carcinogenesis as the result of *RB1* mutations (8%), cyclin dependent kinase inhibitor 2A (*CDKN2A*) mutations and homozygous deletions, or *CDKN2A* promoter methylation [35]. As for *TP53* mutations, *RB1* inactivating mutations may commonly be found in advanced HCC, highlighting a correlation between this molecular alteration and tumor progression [32]. In addition, mutations in *CDKN2A* and *RB1* are enriched in tumors with poor prognosis, suggesting a role of p21 pathway inactivation in tumor aggressiveness [21,44]. Indeed, in 130 patients who underwent liver resection, the expression of p21 was associated with significantly shorter survival [45].

Finally, viral insertions of HBV or AAV2 in Cyclin E1 (*CCNE1*) induced its overexpression and dysregulation of the cell cycle, defining a homogeneous subclass HCC characterized by a rearrangement signature due to the replication stress [25,38,46].

### 2.4. Epigenetic Dysregulation

Epigenetic dysregulation plays an important role in liver carcinogenesis by modifying gene expression through various mechanisms, including chromatin remodeling, histone and methylation modifications. Inactivating mutations of AT-rich interaction domain containing protein 1A (*ARID1A*) and 2 (*ARID2*), which encode key components of SWItch/Sucrose Non-Fermentable (SWI/SNF) chromatin remodeling complexes, have been described in up to 10–15% and 5–8% of HCC cases, respectively, and lead to the repression of genes regulated by the transcription factor E2F [26,35]. *ARID1A* and *ARID2* mutations are found with the same frequency across all BCLC-stages and are more frequent in HCC related to alcohol liver disease [21,32]. Moreover, a positive interaction between *AXIN1* and *ARID1A* and between *CTNNB1* and *ARID2* has been described [21]. Epigenetic alterations involved in liver carcinogenesis also include histone modifications that, consequently, affect DNA accessibility. The acetylation of lysine residues in the histone tails reduces their affinity for DNA, making the latter more accessible to transcription factors and thus modifying gene expression [35]. Lysine residues of histones may also be subject to a methylation process that can either favor or inhibit gene expression. Indeed, genes that belong to the histone methylation writer family (e.g., *MLL2*, *MLL3*, and *MLL4*) and act by adding and removing H3K4 methyl, can be affected by somatic mutations and HBV and AAV2 insertion [26,35]. More generally, changes in DNA methylation gradually increase from cirrhosis, dysplastic nodules and HCC, and aberrant methylation of four gatekeeper genes (testis-specific Y-encoded-like protein 5 (*TSPYL5)*; Potassium Voltage-Gated Channel Subfamily A Member 3 (*KCNA3*); lactate dehydrogenase B (*LDHB*); and serine peptidase inhibitor Kunitz Type 2 *(SPINT2)*) have recently been linked to the transition to early HCC [39,47]. 

### 2.5. Oxidative Stress Pathway

Persistent liver injury due to chronic inflammation and exposure to carcinogens exposed the hepatocytes to oxidative stress. Nuclear factor erythroid 2-related factor 2 (NRF2), encoded by *NFE2L2*, and kelch-like ECH-associated protein 1 (encoded by *KEAP1*) pathway activation plays a key role in protecting cells from oxidative stress. Activating mutations of *NFE2L2* and inactivating mutations of *KEAP1* are found in approximately 6% and 4% of HCC, and confer to cancer cells an advantage of resistance to oxidative stress through the inhibition of KEAP1-mediated degradation of NRF2 [26]. A significant association between *NFE2L2* and *KEAP1* and mutations in *CTNNB1* or *AXIN1* has been described, suggesting that the oxidative stress responses might cooperate with the Wnt/β-catenin pathway in promoting liver carcinogenesis [48]. 

### 2.6. Activation of RAS/RAF/ MAP Kinase and PI3K/AKT/MTOR Pathways

Rapidly Accelerated Fibrosarcoma (RAF)/Rat Sarcoma (RAS)/Mitogen-activated protein (MAP) kinase and the phosphoinositide 3-kinase (PI3K)/protein kinase B (AKT)/mechanistic target of rapamycin (MTOR) pathway are activated in approximately 5–10% of HCCs [26]. RAS proteins recruit RAF that phosphorylate MAP kinases, promoting cell proliferation and inhibition of apoptosis [49]. RAF/RAS/MAP kinase pathway activation could be linked to activating mutations of *RAS* (<1%) or mutations of Ribosomal Protein S6 Kinase (*RPS6KA3*) (2–10%) [32]. Additionally, the RAF/RAS/MAP kinase pathway might also be constitutively activated through the fibroblast growth factor receptor (FGFR) and the vascular endothelial growth factor receptor (VEGFR) [26,50]. Indeed, approximately 6% and 4% of HCC cases have a *FGF19* (locus 11q13) and a *VEGF* (locus 6p21) focal amplification, respectively [51]. Although rare, inactivating mutations of *RPS6KA3* (6%) and amplifications of *FGF19* (5% to 10%) could be targeted using MEK and FGFR4 inhibitors, respectively [32,52,53,54]. Activating mutations of phosphatidylinositol-4,5-bisphosphate 3-kinase, catalytic subunit alpha (*PIK3CA*) (2%), inactivating mutations of *TSC1* or *TSC2* (3–8%) and homozygous deletion of phosphatase and tensin homolog (*PTEN*) (2–3%) favor the permanent activation of the AKT/MTOR pathway [55]. As reported above, *TSC2* mutations often occur together with *TP53* mutations [21]. 

## 3. Molecular Classification of Hepatocellular Carcinoma

Based on genomic, transcriptomic and epigenetic data, HCC could be classified in homogeneous subgroups of tumors groups correlated with clinical features, risk factors and histopathological characteristics [56,57]. 

The “proliferative” class (50% of cases) is composed of poorly differentiated and aggressive tumors with an enrichment of HBV related HCC with a high serum AFP level [56,57]. These tumors are characterized by chromosomal instability, *TP53* inactivating mutations, amplification of *FGF19* and *CCND1*, as well as the activation of pathways involved in cell proliferation and survival (RAS/RAF/MAP kinase and PI3K/AKT/MTOR and Metabolic Equivalent of Task (MET)) [56]. 

The “proliferative” class includes a subgroup of “progenitors” HCCs (G1 transcriptomic class), defined by the overexpression of hepatic progenitor markers (Epithelial cell adhesion molecule (EPCAM), AFP, insulin-like growth factor (IGF) 2) and the occurrence of inactivating mutations of *RPS6KA3* and BRCA1 associated protein-1 (*BAP1*) mutations [56,58]. Interestingly, HCC with *BAP1* mutations harbored fibrolamellar-like features at histology and a dysregulation of the PKA pathway at the transcriptomic level [59]. In addition, the “proliferative” class included HCC of the G3 transcriptomic class that are associated with a poor prognosis, enriched in *TSC1* and *TSC2* mutations and *FGF19*/*CCND1* amplification and characterized by a peculiar histological phenotype known as “macrotrabecular massive”, easily identified by the pathologist at liver biopsy or on surgical sample [24,57,60]. 

The ‘‘non-proliferation” class includes well differentiated and chromosomally stable tumors which usually develop in the context of HCV chronic infection or chronic alcohol consumption [56,57]. The “non-proliferation” class is composed of two different subgroups. The first subgroup is composed of the G5 and G6 transcriptomic classes and is characterized by *CTNNB1* mutations [56]. At the pathological level, these tumors are usually characterized by cholestasis, β-catenin translocation in the nucleus and overexpression of glutamine-synthase, a target gene of the Wnt/b-catenin pathway [57]. Tumors characterized by an activation of canonical Wnt/β -catenin signaling through *CTNNB1* mutations were usually described with a low immune infiltrate, even if recent studies suggested a more heterogeneous profile at the immune level [61]. On the other hand, the second part of the “non-proliferation class” includes the G4 transcriptomic subgroup of HCC that is characterized by a transcriptomic program closed to those mature hepatocytes, and which included steato-hepatitic tumors characterized by the interleukin (IL6)/Janus kinase (JAK)/signal transducers and activators of transcription (STAT) pathway activation [24,57]. 

## 4. Tumor Heterogeneity in HCC

In addition to the inter-patient tumor heterogeneity, intratumoral and intertumoral heterogeneity needs to be taken into account. The term intratumoral heterogeneity refers to the presence within the same lesion of multiple cell populations that exhibit divergent molecular and biological characteristics [62]. After malignant transformation, the parental cancer cell undergoes progressive expansion and the cells originating from this expansion may acquire additional molecular alterations at genetic and epigenetic levels, forming distinct subclones [62]. Thus, from a molecular point of view, within the same tumor we can find “clonal” or “trunk” mutations that are ubiquitously present in all cancer cells, and that are supposed, therefore, to be acquired early during carcinogenesis, and “private”, “branch” or “subclonal” mutations that are acquired only by some of the cancer cells as a result of the different endogenous and/or exogeneous selective pressures [63]. Single cell techniques tackle this issue, also enabling a good characterization of the tumor hepatocytes and their microenvironment that modulates the process of tumorigenesis contributing to tumor diversity [64,65,66,67]. From a therapeutic point of view, it would be more advantageous to use drugs directed against clonal mutations to target all the cells composing the tumor. However, the most frequent clonal mutations in HCC include *TERT* promoter, *CTNNB1*, and *TP53* mutations for which no targeted therapies are yet available [63,68,69]. The term intertumor heterogeneity refers to the differences at the molecular level observed between distinct nodules developed in the same patient. In the case of multifocal HCC, lesions may originate from the same primary tumor, representing intrahepatic metastases, or from a different clone that develops independently on the cirrhotic background [68]. Intrahepatic metastases present a different genomic profile compared with a primary tumor, but this molecular divergence is less than that found in tumors which develop independently [70]. However, most of the studies that have investigated tumor heterogeneity in HCC have been conducted on resected tumors or on transplanted liver [63,68]. Therefore, the impact of tumor heterogeneity in advanced tumors remains to be investigated. The intra- and intertumoral heterogeneity could explain why a single tumor sample may not be precise enough to capture the molecular landscape of HCC and choose the optimal therapeutic strategy. The development of liquid biopsy, such as circulating tumor DNA, has been proposed as a way to bypass the limitations related to tumor heterogeneity.

## 5. Circulating Tumor DNA in Hepatocellular Carcinoma

### 5.1. General Considerations on Liquid Biopsy and Circulating Tumor DNA

**Figure 2:**
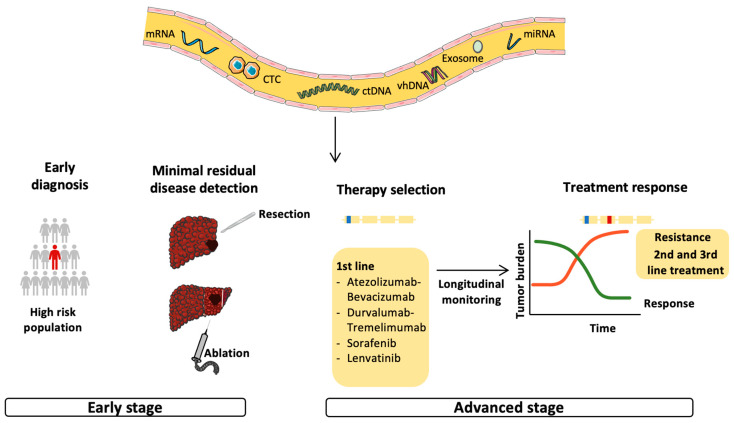
Possible applications of circulating tumor DNA in the management of HCC. Circulating tumor DNA (ctDNA) reflects tumor burden and has been tested for early cancer detection and minimal residual disease (MRD) monitoring in the early stage of HCC. In addition, ctDNA should be evaluated for the identification of targetable mutations and biomarkers and, therefore, be used for therapy selection. The non-invasiveness and the ability to reflect the patient’s oncological picture in real time makes ctDNA an ideal tool for longitudinal monitoring and early detection of acquired resistance, leading to a switch to second- or third-line therapy.

The term liquid biopsy refers to the different tumor components released in biological fluids, such as circulating tumor nucleic acids, circulating tumor cells (CTCs), mRNA, microRNA and exosomes (Figure 2) [71,72]. CtDNA represents only a small percentage of the cellular free-DNA (cfDNA) [73]. CtDNA is a double-stranded DNA fragment of about 150 bp in length, slightly less than those of cfDNA [72], and is passively released into the bloodstream via apoptosis or the necrosis of tumor cells or CTCs, or via active secretion by tumor cells [72]. The half-life of ctDNA is short (<2 h) and, therefore, it reflects the patient’s oncological picture in real time [73] and could be used for the longitudinal monitoring of disease progression and response to therapy [74]. It is also an non-invasive tool, useful for serial assessment using a simple blood sample, and could help to define the mutation profile of tumors when tissue is insufficient or unavailable [72,74]. For this reason, the use of ctDNA could be particularly useful in HCC for which the diagnosis may not require tumor biopsy in cirrhotic patients in the presence of typical radiological features [15]. To note, most studies performed in the field have not analyzed the concomitant HCC, impairing the ability to assess the sensitivity and specificity of ctDNA to identify the genetic alterations present in the tumor. In contrast to non-small cell lung [75] and breast cancers [76], the use of ctDNA in clinical practice has not yet been approved for HCC, although some studies have evaluated its role for early diagnosis, early detection of recurrence and monitoring patients under systemic treatments. The principal studies evaluating the role of ctDNA in HCC are summarized in Table 1. 

### 5.2. Early Diagnosis

The use of ctDNA as a screening tool is not yet approved for any malignancy, although some tests have been tested for this purpose. CancerSEEK is a blood test based on the assessment of the levels of circulating proteins and mutations in cell-free DNA evaluated in eight different cancers, including liver cancer, and has reported a high sensitivity and specificity to detect these types of cancer [102]. Additionally, the methylation profiling of ctDNA through whole-genome bisulfite sequencing showed good results for early cancer detection and in determining the tissue of origin of the tumor [103]. Kisiel et al., 2019, identified a combination of six plasma methylated DNA markers that accurately detect HCC across all BCLC stages [81], and the role of circulating tumor DNA methylation markers for the diagnosis and prognosis of HCC has been also described in a large cohort of 1098 HCC patients [82]. 

However, early diagnosis of cancers may be difficult due to the low levels of ctDNA detected in bodily fluids, as this level is usually linked with tumor burden [72]. In HCC, one study reported that cfDNA concentration may identify tumors in HCV-positive patients with good sensitivity and specificity values [77]. Furthermore, various groups have combined ctDNA concentration with other biomarkers, such as AFP [78] and DCP [79], in order to increase its diagnostic accuracy (85% sensitivity and 93% specificity to distinguish patients with HCC from those without) [79]. 

Even if these studies seem promising, more data are required, using prospective cohorts of patients in order to validate their diagnostic accuracy specifically in very early (BCLC 0) and early HCC (BCLC A) that is the target for early detection in clinical practice.

### 5.3. Prognostic Tool

After curative treatment, ctDNA is supposed to completely disappear. Consequently, a detectable ctDNA or a subsequent reappearance after an initial negativation is usually linked with a high risk of tumor recurrence. For the above reasons, ctDNA may represent the ideal method to monitor patients after curative treatments in order to detect the presence of minimal residual disease and predict tumor recurrence [73]. Women with triple-negative breast cancer undergoing neoadjuvant treatment seem to have a worse prognosis if ctDNA is persistently detectable during therapy [104]. The role of post-surgery ctDNA has been also proposed to guide the adjuvant treatment and monitor its efficacy [105,106]. Tie et al., 2022, compared the recurrence free survival of stage II colon cancer patients who were assigned to adjuvant treatment, using ctDNA results at four or seven weeks after surgery or standard clinicopathological features demonstrating that ctDNA guided management was non inferior compared to the standard of care [105]. Moreover, Henriksen et al., 2022, observed that patients with stage III colorectal cancer with post-adjuvant therapy detectable ctDNA had a shorter relapse-free survival compared with patients who had a permanent clearance [106]. Independently of the realization of an adjuvant treatment, the detection of ctDNA after surgery has been linked to a worse prognosis being able to indicate minimal residual disease. In patients with stage I-III non-small cell lung cancer, Chaudhuri et al., 2017, observed that ctDNA detection after four months of curative treatment was associated with a shorter disease-free survival and that ctDNA detection could identify tumor recurrence before imaging with a median of 5.2 months [107]. Similar results have also been reported for breast [108], rectal [109] and gastric cancer [110]. 

The persistence of elevated ctDNA concentrations after liver resection were related to a poor survival, with a higher risk of metastasis also in HCV-related HCC [88]. In addition, Cai et al., 2019, demonstrated that the serial monitoring of postoperative ctDNA was able to detect 59% of patients with early recurrence [89]. However, the implemental value compared to classical follow-up by imaging remains to be demonstrated in HCC patients. Moreover, additional studies showed that the prognostic role of ctDNA was not only related to its concentration but also to the mutational profile [90] and the variant allele frequency (VAF) [91]. Li et al., 2020, proposed, in HBV-related HCC, the use of virus–host chimera DNA (vhDNA) to detect minimal residual disease and monitor recurrence after resection [92]. They observed that, in 23.3% of patients who underwent liver resection, the same vh-DNA signature was detectable in plasma samples collected two months after the treatment, and that in 90% of the cases an HCC recurrence occurred within one year [92]. Finally, the role of adjuvant therapy in HCC patients with a detectable ctDNA after curative treatments should be tested in future clinical trials.

### 5.4. Monitoring of Systemic Treatments

CtDNA could be used as a theranostic tool in order to detect targetable genetic alterations in the blood of patients with advanced cancer, especially when tumor samples are not available [111,112]. In addition, a drop in ctDNA levels during systemic treatment has been associated with better prognosis [113]. To date, no biomarkers able to identify HCC patients who are more likely to respond to the different treatments have been validated, except for high serum AFP level below 400 ng/mL to guide treatment with ramucirumab [114]. 

In one study, the pretreatment cfDNA levels and copy number alteration assessed by low depth whole-genome sequencing of cfDNA predicts the outcome of patients with HCC receiving sorafenib [94]. Preliminary data in patients undergoing systemic therapy or transarterial chemoembolization showed that the presence of ctDNA of *TERT* promoter mutation, especially when VAF is greater than 0.01, were associated with a poor prognosis [95]. Moreover, the combination of *TERT* promoter mutation and serum AFP, in addition to ctDNA concentration, was associated with a poor survival in 85 patients treated with atezolizumab-bevacizumab [96]. Finally, a reduction in the VAF, four weeks after the beginning of the treatment, has been associated with a longer progression free survival in 24 patients with HCC treated with lenvatinib [97]. 

Another important potential role of ctDNA is the detection of secondary (acquired) resistance to treatment, even before radiological evaluation, enabling a rapid switch in therapy strategy and the identification of new therapeutic targets [115,116]. In the case of Non-Small Cell Lung Cancer (NSCLC), the presence of the Epithelial Growth Factor Receptor (*EGFR*) T790M mutation in ctDNA may be detectable months before radiological progression and can be used to switch from EGFR tyrosine kinase inhibitors (TKIs) to osimertinib [117]. Similarly, the detection of estrogen signaling receptor (*ESR1*) mutation in patients with breast cancer treated with aromatase inhibitors can be detected months before progression [118]. Recent data showed that ctDNA analysis can be used to guide anti-EGFR rechallenge in patients with metastatic colorectal cancer, while proposing a different therapy for those with persistent detectable mutations in the ctDNA of genes belonging to EGFR downstream effectors or EGFR extracellular domain [119]. 

In a small cohort of 23 patients with HCC receiving tyrosine kinase inhibitors, the development of alterations in the AKT/mTOR pathway was correlated with a reduced progression free survival [98]. Finally, more data are needed on patients with advanced HCC under atezolizumab/bevacizumab and durvalumab/tremelimumab, including longitudinal analysis of ctDNA.

## 6. Conclusion and Future Perspectives

Our knowledge of the HCC molecular landscape has progressively improved, but its translation into clinical practice remains an unmet need. Unlike other malignancies, no molecular alteration guided therapy is currently available for HCC, or for biomarkers that predict the response or resistance to systemic treatments, with the exception of ramucirumab [114]. The implementation of liver biopsy in a randomized controlled trial and in prospective cohorts of patients will help to the identification of the mechanisms of primary and secondary resistance to systemic therapies, overcoming the current absence of biomarkers. CtDNA represents an emerging tool for screening, early detection and prognosis and could overcome the problems related to tissue sampling, such as tumor heterogeneity or accessibility. Moreover, the fact that ctDNA is a non-invasive biomarker could allow longitudinal monitoring, enabling a rapid recognition of response or progression under treatment even before imaging evaluation. Including ctDNA analysis to tissue samples in clinical trials in the field of HCC will be critical in establishing its utility in future clinical practice.

## Figures and Tables

**Table 1:** Major studies describing the role of ctDNA in early diagnosis, predicting prognosis and monitoring systemic treatment in HCC.

	Study	Input	Target	Patients		Main Results
Early diagnosis	Iizuka et al. [77]	7 mL blood (serum)	Total amount cfDNA	52 HCC (46% TNM I stage) 30 HCV chronic hepatitis 16 healthy controls	Asia	Sens. 69.2% (AFP 69.2%, DCP 73.1%) Spec. 93.3% (AFP 72.7%, DCP 75%) AUC 0.79 (AFP 0.79, DCP 0.73)
	Yan et al. [78]	Plasma	Total amount cfDNA, AFP	24 HCC 62 HBV chronic hepatitis	Asia	Sens. 87% (cfDNA 62.5%, AFP 47.8%) Spec. 100% (cfDNA 93.6%, AFP 93.2%) AUC 0.98 (cfDNA 0.82, AFP 0.67)
	Qu et al. [79]	2.5 mL plasma	*TP53*, *CTNNB1*, *AXIN 1*, *TERT*, HBV insertion site, AFP, DCP, age, sex	65 HCC, 70 CLD (training) 24 HCC, 307 CLD (validation)	Asia	Sens. 85% (training) 100% (validation) Spec. 93% (training), 94% (validation) AUC 0.93 (training)
	Tao et al. [80]	2.0 mL plasma	SCNA	108 HCC (67.6% BCLC 0/A), 101 HBV (training) 89 HCC (100% BCLC0/A), 86 HBV (validation)	Asia	Sens. 70% early Spec. 95% AUC * 0.87 (training), 0.81–0.92 (validation)
	Kisiel et al. [81]	Tissue and ≥1 mL plasma	*HOXA1*, *EMX1*, *AK055957*, *ECE1*, *PFKP* and *CLEC11A*	95 HCC (BCLC 0/A 48%), 51 cirrhosis, 98 healthy controls (phase II study)	USA	Sens. 91% * Spec. 92% * AUC 0.94 (AFP 0.80)
	Xu et al. [82]	1.5 mL plasma	*BMPR1A*, *PSD*, *ARHGAP25*, *KLF3*, *PLAC8*, *ATXN1*, Chr 6:170, Chr 6:3, *ATAD2*, Chr 8:20	715 HCC, 560 healthy controls (training) 383 HCC, 275 healthy controls (validation), 16% TNM I stage (training and validation)	Asia	Sens. 86% (training), 83% (validation) Spec. 94% (training), 91% (validation) AUC 0.97 (training), 0.94 (validation), 0.82 (AFP)
	Chalasani et al. [83]	3 mL plasma	*HOXA*, *EMX1*, *TSPYL5*, *B3GALT6*, AFP, AFP-L3	135 HCC (56.2% BCLC 0/A) 302 controls (87% cirrhotic)	USA (95%), Europe, Asia	Sens. 71% * (GALAD 58% *, AFP 21% *) Spec. 90% * (GALAD 86% *, AFP 98%*) AUC 0.92 (GALAD 0.87, AFP 0.81)
	Oussalah et al. [84]	3.5 mL plasma	*SEPT9*	51 HCC, 135 CLD (training) 47 HCC, 56 CLD (validation) 25.5% BCLC0/A (training and validation cohort)	Europe	Sens. 94.1% (training), 85% (validation) Spec. 84% (training), 91% (validation) AUC 0.94 (training), 0.93 (validation), 0.85 (AFP)
	Cai et al. [85]	5–10 mL blood	5-hmC 32-gene panel	335 HCC (100% BCLC 0/A), 263 cirrhosis, 522 healthy controls (training) 809 HCC (27% BCLC 0/A), 129 cirrhosis, 256 healthy controls (validation)	Asia	Sens. 89.6% (training), 82.7% (validation) Spec. 78.9% (training), 76.4% (validation) AUC ^1^ 0.87 (training), 0.85 (validation) AFP 0.79 (training), 0.69 (validation)
	Koto et al. [86]	0.4 mL serum, tissue	*SEPT9*	136 HCC (45% BCLC 0/A)45 CLD80 healthy controls	Asia	Sens. 63.2%Spec. 90%AUC 0.81
	Lin et al. [87]	plasma	28 methylation markers	122 HCC (37% early stage) 125 CLD (37% cirrhosis)	Asia	Sens. 76% ^ (AFP 57% ^, GALAD 65% ^) Spec. 91% (AFP 97%, GALAD 94%) AUC 0.94 (AFP 0.85, GALAD 0.90)
Minimal residual disease detection	Tokuhisa et al. [88]	1 mL serum, Real-time qPCR	Total amount cfDNA	96 resected HCC patients 100 HCV chronic hepatitis	Asia	cfDNA levels associated with poorer OS (HR 3.4) and higher risk of metastases (OR 4.5)
	Cai et al. [89]	Serum, plasma, tissue	CNV, SNV, AFP, AFP-L3 and DCP	34 resected HCC patients	Asia	ctDNA mutations detected after surgery (90 days) independently associated with poorer RFS (*p* = 0.001) and OS (0.001). DCP independently associated with OS (*p* = 0.019). ctDNA and DCP combination increased MRD detection.
	García-Fernández et al. [90]	-	*TP53*	24 transplanted patients	Europe	*TP53* could be used as a biomarker of tumor recurrence
	Zhu et al. [91]	4 mL plasma, tissue	WES	41 resected HCC patients	Asia	Sustained ctDNA positivity (1 week–4 months) associated with higher risk of tumor recurrence; baseline VAF strong independent predictor of RFS
	Li et al. [92]	1 mL plasma, tissues	vh-chimera DNA	50 resected HCC	Asia	Vh-DNA detection (2 months) independent factor of 1 year recurrence (HR 4.66)
	Ako et al. [93]	1 mL plasma, tissues	*TERT*	36 resected HCC	Asia	*TERT* positive patients had poorer RFS (*p* = 0.02). *TERT* promoter mutations significant predictor of RFS (HR 3.1)
Systemic treatment response	Oh et al. [94]	1.5 mL plasma	Total amount cfDNA, genomic instability, VEGFA amplification	151 patients who received first-line sorafenib 14 healthy controls	Asia	Higher cfDNA levels and genomic instability associated with shorter PFS (HR 1.7 and 2.09, respectively), shorter OS (HR 3.5 and 3.35, respectively). VEGFA ratio not associated with outcome.
	Hirai et al. [95]	1 mL plasma	*TERT*	130 HCC undergoing TKI or TACE	Asia	*TERT* promoter mutations associated with poorer OS aHR = 1.94, higher fractional abundance associated with worse OS
	Matsumae et al. [96]	Plasma	Total amount cfDNA, 25 genes	85 patients who received atezolizumab-bevacizumab	Asia	ctDNA detection associated with shorter PFS. *CTNNB1* mutations not associated with treatment response or prognosis. *TERT* mutations and AFP independent predictors of worse OS.
	Fujii et al. [97]	2 mL plasma	74 genes	24 patients receiving lenvatinib	Asia	VAF_mean_ reduction associated with longer PFS and reduction in tumor burden
	Von Felden et al. [98]	Plasma, tissue	25 genes	26 HCC, 10 CLD (technical validation) 77 HCC (BCLC C 73%; 23 underwent TKI, 38 underwent CPI), 8 CLD (clinical cohort)	USA	Mutations in the PI3K/MTOR pathway associated with sorter PFS in patients undergoing TKI (*p* < 0.001). Wnt pathway mutations not associated with PFS, OS or response in patients undergoing CPI. Increase in VAF associated with resistance for both TKI and CPI.
	Nakatsuka et al. [99]	1 mL plasma	Total amount cfDNA, *TERT*	100 HCC patients treated by RFA, TACE, systemic treatment	Asia	Difference between cfDNA before and after systemic treatment predicted tumor response (AUC 0.807)
	Alunni-Fabbroni et al. [100]	5 mL blood (plasma)	Total amount cfDNA, 597 cancer genes	13 HCC (SORAMIC trial)	Europe	cfDNA during treatment associated with worse outcome (improved sensitivity over AFP). Dynamic change of mutation status correlated with treatment response
	Ikeda et al. [101]	10 mL blood (plasma)	68 cancer genes	14 advanced HCC	Asia	Treatment oriented according to genomic alterations found in ctDNA in two patients with good treatment responses: - *CDKN2A* and *CTNNB1* treated by palbociclib and celecoxib - *MET*, *TP53* and *PTEN* treated by sirolimus and cabozantinib

* BCLC 0/A stage, ^1^ HCC versus CLD HCC: hepatocellular carcinoma, ^ AJCC stage I–II. aHR: adjusted hazard ratio; cfDNA: circulating free DNA; CPI: immune checkpoint inhibitors; CLD: chronic liver disease, CNV: copy number variation; ctDNA: circulating tumor DNA; DCP: des-gamma-carboxy prothrombin; HR: hazard ratio; LR: liver resection; MRD: minimal residual disease; OR: odds ratio; OS: overall survival; PBMC: peripheral blood mononuclear cells; PFS: progression free survival; RFA: radiofrequency ablation; RFS: relapse free survival; SCNA: somatic copy number aberrations; SNV: single nucleotide variation; TACE: transarterial chemoembolization; TNM: tumor node metastasis, TKI: tyrosine-kinase inhibitor; VAF: variant allele frequency; Vh: virus–host; WES: whole exome sequencing.

## Abbreviations

aHR: adjusted hazard ratio; AAV2: adeno-associated virus type 2; AFP: alpha-foetoprotein; AKT: protein kinase B; APC: adenomatous polyposis coli; ARID1A: AT-rich interaction domain containing protein 1A; BAP-1: BRCA1 associated protein-1; BCLC: Barcelona Clinic Liver Classification; CCND1: Cyclin D1; CCNE1: Cyclin E; cfDNA: cellular free-DNA; CDKN2A: cyclin dependent kinase inhibitor 2A; CLD: chronic liver disease; CNV: copy number variation; CPI: immune checkpoint inhibitors; ctDNA: circulating tumor DNA; CTNNB1: catenin beta 1; DCP: des-gamma-carboxy prothrombin; EPCAM: Epithelial cell adhesion molecule; EGFR: Epithelial Growth Factor Receptor; ESR: estrogen signaling receptor; FGF: fibroblast growth factor; FGFR: fibroblast growth factor receptor; GSK3B: glycogen synthase kinase 3; HBV: hepatitis B virus; HCV: hepatitis C virus; HCC: hepatocellular carcinoma; HGDN: high-grade dysplastic nodule; HSD17B13: 17β-Hydroxysteroid dehydrogenase 13; HR: hazard ratio; IGF: insulin-like growth factor; KEAP1: Kelch-like ECH-associated protein 1; KCNA3: Potassium Voltage-Gated Channel Subfamily A Member 3; LDHB: lactate dehydrogenase B; LGDN: low-grade dysplastic nodule; LR: liver resection; MAP: Mitogen-activated protein; MBOAT7: Membrane-bound O-acyltransferase domain containing 7; MET: Metabolic Equivalent of Task; MRD: minimal residual disease; MTOR: mechanistic target of rapamycin; NAFLD: non-alcoholic fatty liver disease; NFE2L2: Nuclear factor erythroid 2-related factor 2; NGS: next-generation sequencing; OR: odds ratio; OS: overall survival; PBMC: peripheral blood mononuclear cells; PI3K: phosphoinositide 3-kinase; PIK3CA: phosphatidylinositol-4,5-bisphosphate 3-kinase, catalytic subunit alpha; PFS: progression free survival; PNPLA3: Patatin-like phospholipase domain-containing protein 3; PTEN: phosphatase and tensin homolog; RAF: Rapidly Accelerated Fibrosarcoma; RAS: Rat Sarcoma;RB: retinoblastoma; RFA: radiofrequency ablation; RFS: relapse free survival; RPS6KA3: Ribosomal Protein S6 Kinase; SCNA: somatic copy number aberrations; SLC12A7: Solute Carrier Family 12 Member 7; SLC7A2: Solute Carrier Family 7 Member 2; SNP: single nucleotide polymorphisms; SNV: single nucleotide variation; SPINT2: serine peptidase inhibitor Kunitz Type 2; SWI/SNF: SWItch/Sucrose Non-Fermentable; TACE: transarterial chemoembolization; TERC: telomerase RNA component; TERT: telomerase reverse transcriptase; TKI: tyrosine-kinase inhibitor; TM6SF2: Transmembrane 6, superfamily member 2; TNM: tumor node metastasis; TP53: tumor protein 53; TSPYL5: testis-specific Y-encoded-like protein 5; TSC2: Tuberous Sclerosis Complex; 2VAF: variant allele frequency; VEGFR: vascular endothelial growth factor receptor; Vh: virus–host; vh-DNA: virus–host chimera DNA; WES: whole exome sequencing; WNT3A-WNT9A: Wnt Family Member 3A and 9A, YAP: yes-associated protein 1.

## References

[1] Llovet J.M., Kelley R.K., Villanueva A., Singal A.G., Pikarsky E., Roayaie S., Lencioni R., Koike K., Zucman-Rossi J., Finn R.S. (2021). Hepatocellular Carcinoma. Nat. Rev. Dis. Prim..

[2] Falleti E., Fabris C., Cmet S., Cussigh A., Bitetto D., Fontanini E., Fornasiere E., Bignulin S., Fumolo E., Bignulin E. (2011). PNPLA3 Rs738409C/G Polymorphism in Cirrhosis: Relationship with the Aetiology of Liver Disease and Hepatocellular Carcinoma Occurrence: PNPLA3 Rs738409polymorphism and Liver Cancer. Liver Int..

[3] Trépo E., Nahon P., Bontempi G., Valenti L., Falleti E., Nischalke H.-D., Hamza S., Corradini S.G., Burza M.A., Guyot E. (2014). Association between the *PNPLA3* (Rs738409 C>G) Variant and Hepatocellular Carcinoma: Evidence from a Meta-Analysis of Individual Participant Data. Hepatology.

[4] Burza M.A., Pirazzi C., Maglio C., Sjöholm K., Mancina R.M., Svensson P.-A., Jacobson P., Adiels M., Baroni M.G., Borén J. (2012). PNPLA3 I148M (Rs738409) Genetic Variant Is Associated with Hepatocellular Carcinoma in Obese Individuals. Dig. Liver Dis..

[5] Liu Y.-L., Patman G.L., Leathart J.B.S., Piguet A.-C., Burt A.D., Dufour J.-F., Day C.P., Daly A.K., Reeves H.L., Anstee Q.M. (2014). Carriage of the PNPLA3 Rs738409 C >G Polymorphism Confers an Increased Risk of Non-Alcoholic Fatty Liver Disease Associated Hepatocellular Carcinoma. J. Hepatol..

[6] Seko Y., Sumida Y., Tanaka S., Mori K., Taketani H., Ishiba H., Hara T., Okajima A., Umemura A., Nishikawa T. (2017). Development of Hepatocellular Carcinoma in Japanese Patients with Biopsy-Proven Non-Alcoholic Fatty Liver Disease: Association between PNPLA3 Genotype and Hepatocarcinogenesis/Fibrosis Progression: PNPLA3 Genotype and HCC in NAFLD. Hepatol. Res..

[7] Newberry E.P., Hall Z., Xie Y., Molitor E.A., Bayguinov P.O., Strout G.W., Fitzpatrick J.A.J., Brunt E.M., Griffin J.L., Davidson N.O. (2021). Liver-Specific Deletion of Mouse Tm6sf2 Promotes Steatosis, Fibrosis, and Hepatocellular Cancer. Hepatology.

[8] Liu Y.-L., Reeves H.L., Burt A.D., Tiniakos D., McPherson S., Leathart J.B.S., Allison M.E.D., Alexander G.J., Piguet A.-C., Anty R. (2014). TM6SF2 Rs58542926 Influences Hepatic Fibrosis Progression in Patients with Non-Alcoholic Fatty Liver Disease. Nat. Commun..

[9] Yang J., Trépo E., Nahon P., Cao Q., Moreno C., Letouzé E., Imbeaud S., Gustot T., Deviere J., Debette S. (2019). PNPLA3 and TM6SF2 Variants as Risk Factors of Hepatocellular Carcinoma across Various Etiologies and Severity of Underlying Liver Diseases. Int. J. Cancer.

[10] Donati B., Dongiovanni P., Romeo S., Meroni M., McCain M., Miele L., Petta S., Maier S., Rosso C., De Luca L. (2017). MBOAT7 Rs641738 Variant and Hepatocellular Carcinoma in Non-Cirrhotic Individuals. Sci. Rep..

[11] Yang J., Trépo E., Nahon P., Cao Q., Moreno C., Letouzé E., Imbeaud S., Bayard Q., Gustot T., Deviere J. (2019). A 17-Beta-Hydroxysteroid Dehydrogenase 13 Variant Protects From Hepatocellular Carcinoma Development in Alcoholic Liver Disease. Hepatology.

[12] Abul-Husn N.S., Cheng X., Li A.H., Xin Y., Schurmann C., Stevis P., Liu Y., Kozlitina J., Stender S., Wood G.C. (2018). A Protein-Truncating *HSD17B13* Variant and Protection from Chronic Liver Disease. N. Engl. J. Med..

[13] Trépo E., Caruso S., Yang J., Imbeaud S., Couchy G., Bayard Q., Letouzé E., Ganne-Carrié N., Moreno C., Oussalah A. (2022). Common Genetic Variation in Alcohol-Related Hepatocellular Carcinoma: A Case-Control Genome-Wide Association Study. Lancet Oncol..

[14] Siegel R.L., Miller K.D., Fuchs H.E., Jemal A. (2022). Cancer Statistics, 2022. CA A Cancer J. Clin..

[15] Galle P.R., Forner A., Llovet J.M., Mazzaferro V., Piscaglia F., Raoul J.-L., Schirmacher P., Vilgrain V. (2018). EASL Clinical Practice Guidelines: Management of Hepatocellular Carcinoma. J. Hepatol..

[16] Omata M., Cheng A.-L., Kokudo N., Kudo M., Lee J.M., Jia J., Tateishi R., Han K.-H., Chawla Y.K., Shiina S. (2017). Asia–Pacific Clinical Practice Guidelines on the Management of Hepatocellular Carcinoma: A 2017 Update. Hepatol. Int..

[17] Marrero J.A., Kulik L.M., Sirlin C.B., Zhu A.X., Finn R.S., Abecassis M.M., Roberts L.R., Heimbach J.K. (2018). Diagnosis, Staging, and Management of Hepatocellular Carcinoma: 2018 Practice Guidance by the American Association for the Study of Liver Diseases: Marrero et Al. Hepatology.

[18] U.S. Food & Drug Administration FDA Approves Tremelimumab in Combination with Durvalumab for Unresectable Hepatocellular Carcinoma. https://www.fda.gov/drugs/resources-information-approved-drugs/fda-approves-tremelimumab-combination-durvalumab-unresectable-hepatocellular-carcinoma.

[19] Reig M., Forner A., Rimola J., Ferrer-Fàbrega J., Burrel M., Garcia-Criado Á., Kelley R.K., Galle P.R., Mazzaferro V., Salem R. (2022). BCLC Strategy for Prognosis Prediction and Treatment Recommendation: The 2022 Update. J. Hepatol..

[20] Nault J., Villanueva A. (2021). Biomarkers for Hepatobiliary Cancers. Hepatology.

[21] Schulze K., Imbeaud S., Letouzé E., Alexandrov L.B., Calderaro J., Rebouissou S., Couchy G., Meiller C., Shinde J., Soysouvanh F. (2015). Exome Sequencing of Hepatocellular Carcinomas Identifies New Mutational Signatures and Potential Therapeutic Targets. Nat. Genet..

[22] Totoki Y., Tatsuno K., Covington K.R., Ueda H., Creighton C.J., Kato M., Tsuji S., Donehower L.A., Slagle B.L., Nakamura H. (2014). Trans-Ancestry Mutational Landscape of Hepatocellular Carcinoma Genomes. Nat. Genet..

[23] Ally A., Balasundaram M., Carlsen R., Chuah E., Clarke A., Dhalla N., Holt R.A., Jones S.J.M., Lee D., Ma Y. (2017). Comprehensive and Integrative Genomic Characterization of Hepatocellular Carcinoma. Cell.

[24] Rebouissou S., Nault J.-C. (2020). Advances in Molecular Classification and Precision Oncology in Hepatocellular Carcinoma. J. Hepatol..

[25] Péneau C., Imbeaud S., La Bella T., Hirsch T.Z., Caruso S., Calderaro J., Paradis V., Blanc J.-F., Letouzé E., Nault J.-C. (2022). Hepatitis B Virus Integrations Promote Local and Distant Oncogenic Driver Alterations in Hepatocellular Carcinoma. Gut.

[26] Zucman-Rossi J., Villanueva A., Nault J.-C., Llovet J.M. (2015). Genetic Landscape and Biomarkers of Hepatocellular Carcinoma. Gastroenterology.

[27] Shampay J., Szostak J.W., Blackburn E.H. (1984). DNA Sequences of Telomeres Maintained in Yeast. Nature.

[28] Günes C., Rudolph K.L. (2013). The Role of Telomeres in Stem Cells and Cancer. Cell.

[29] Nault J.-C., Ningarhari M., Rebouissou S., Zucman-Rossi J. (2019). The Role of Telomeres and Telomerase in Cirrhosis and Liver Cancer. Nat. Rev. Gastroenterol. Hepatol..

[30] Ningarhari M., Caruso S., Hirsch T.Z., Bayard Q., Franconi A., Védie A.-L., Noblet B., Blanc J.-F., Amaddeo G., Ganne N. (2021). Telomere Length Is Key to Hepatocellular Carcinoma Diversity and Telomerase Addiction Is an Actionable Therapeutic Target. J. Hepatol..

[31] Wood L.D., Heaphy C.M., Daniel H.D.-J., Naini B.V., Lassman C.R., Arroyo M.R., Kamel I.R., Cosgrove D.P., Boitnott J.K., Meeker A.K. (2013). Chromophobe Hepatocellular Carcinoma with Abrupt Anaplasia: A Proposal for a New Subtype of Hepatocellular Carcinoma with Unique Morphological and Molecular Features. Mod. Pathol..

[32] Nault J., Martin Y., Caruso S., Hirsch T.Z., Bayard Q., Calderaro J., Charpy C., Copie-Bergman C., Ziol M., Bioulac-Sage P. (2020). Clinical Impact of Genomic Diversity From Early to Advanced Hepatocellular Carcinoma. Hepatology.

[33] Nault J.C., Mallet M., Pilati C., Calderaro J., Bioulac-Sage P., Laurent C., Laurent A., Cherqui D., Balabaud C., Zucman-Rossi J. (2013). High Frequency of Telomerase Reverse-Transcriptase Promoter Somatic Mutations in Hepatocellular Carcinoma and Preneoplastic Lesions. Nat. Commun..

[34] Nault J.C., Calderaro J., Di Tommaso L., Balabaud C., Zafrani E.S., Bioulac-Sage P., Roncalli M., Zucman-Rossi J. (2014). Telomerase Reverse Transcriptase Promoter Mutation Is an Early Somatic Genetic Alteration in the Transformation of Premalignant Nodules in Hepatocellular Carcinoma on Cirrhosis. Hepatology.

[35] Dhanasekaran R., Nault J.-C., Roberts L.R., Zucman-Rossi J. (2019). Genomic Medicine and Implications for Hepatocellular Carcinoma Prevention and Therapy. Gastroenterology.

[36] Zhao L.-H., Liu X., Yan H.-X., Li W.-Y., Zeng X., Yang Y., Zhao J., Liu S.-P., Zhuang X.-H., Lin C. (2016). Genomic and Oncogenic Preference of HBV Integration in Hepatocellular Carcinoma. Nat. Commun..

[37] Cevik D. (2015). Common Telomerase Reverse Transcriptase Promoter Mutations in Hepatocellular Carcinomas from Different Geographical Locations. WJG.

[38] Nault J.-C., Datta S., Imbeaud S., Franconi A., Mallet M., Couchy G., Letouzé E., Pilati C., Verret B., Blanc J.-F. (2015). Recurrent AAV2-Related Insertional Mutagenesis in Human Hepatocellular Carcinomas. Nat. Genet..

[39] Desjonqueres E., Campani C., Marra F., Zucman-Rossi J., Nault J. (2022). Preneoplastic Lesions in the Liver: Molecular Insights and Relevance for Clinical Practice. Liver Int..

[40] Nault J.-C., Couchy G., Balabaud C., Morcrette G., Caruso S., Blanc J.-F., Bacq Y., Calderaro J., Paradis V., Ramos J. (2017). Molecular Classification of Hepatocellular Adenoma Associates With Risk Factors, Bleeding, and Malignant Transformation. Gastroenterology.

[41] Abitbol S., Dahmani R., Coulouarn C., Ragazzon B., Mlecnik B., Senni N., Savall M., Bossard P., Sohier P., Drouet V. (2018). AXIN Deficiency in Human and Mouse Hepatocytes Induces Hepatocellular Carcinoma in the Absence of β-Catenin Activation. J. Hepatol..

[42] Rivlin N., Brosh R., Oren M., Rotter V. (2011). Mutations in the P53 Tumor Suppressor Gene: Important Milestones at the Various Steps of Tumorigenesis. Genes Cancer.

[43] Bressac B., Kew M., Wands J., Ozturk M. (1991). Selective G to T Mutations of P53 Gene in Hepatocellular Carcinoma from Southern Africa. Nature.

[44] Ahn S., Jang S.J., Shim J.H., Kim D., Hong S., Sung C.O., Baek D., Haq F., Ansari A.A., Lee S.Y. (2014). Genomic Portrait of Resectable Hepatocellular Carcinomas: Implications of *RB1* and *FGF19* Aberrations for Patient Stratification. Hepatology.

[45] Marhenke S., Buitrago-Molina L.E., Endig J., Orlik J., Schweitzer N., Klett S., Longerich T., Geffers R., Sánchez Muñoz A., Dorrell C. (2014). P21 Promotes Sustained Liver Regeneration and Hepatocarcinogenesis in Chronic Cholestatic Liver Injury. Gut.

[46] Bayard Q., Meunier L., Peneau C., Renault V., Shinde J., Nault J.-C., Mami I., Couchy G., Amaddeo G., Tubacher E. (2018). Cyclin A2/E1 Activation Defines a Hepatocellular Carcinoma Subclass with a Rearrangement Signature of Replication Stress. Nat. Commun..

[47] Hernandez-Meza G., von Felden J., Gonzalez-Kozlova E.E., Garcia-Lezana T., Peix J., Portela A., Craig A.J., Sayols S., Schwartz M., Losic B. (2021). DNA Methylation Profiling of Human Hepatocarcinogenesis. Hepatology.

[48] Guichard C., Amaddeo G., Imbeaud S., Ladeiro Y., Pelletier L., Maad I.B., Calderaro J., Bioulac-Sage P., Letexier M., Degos F. (2012). Integrated Analysis of Somatic Mutations and Focal Copy-Number Changes Identifies Key Genes and Pathways in Hepatocellular Carcinoma. Nat. Genet..

[49] Whittaker S., Marais R., Zhu A.X. (2010). The Role of Signaling Pathways in the Development and Treatment of Hepatocellular Carcinoma. Oncogene.

[50] Moon H., Ro S.W. (2021). MAPK/ERK Signaling Pathway in Hepatocellular Carcinoma. Cancers.

[51] Caruso S., O’Brien D.R., Cleary S.P., Roberts L.R., Zucman-Rossi J. (2021). Genetics of Hepatocellular Carcinoma: Approaches to Explore Molecular Diversity. Hepatology.

[52] Sawey E.T., Chanrion M., Cai C., Wu G., Zhang J., Zender L., Zhao A., Busuttil R.W., Yee H., Stein L. (2011). Identification of a Therapeutic Strategy Targeting Amplified FGF19 in Liver Cancer by Oncogenomic Screening. Cancer Cell.

[53] Caruso S., Calatayud A.-L., Pilet J., La Bella T., Rekik S., Imbeaud S., Letouzé E., Meunier L., Bayard Q., Rohr-Udilova N. (2019). Analysis of Liver Cancer Cell Lines Identifies Agents With Likely Efficacy Against Hepatocellular Carcinoma and Markers of Response. Gastroenterology.

[54] Lim H.Y., Merle P., Weiss K.H., Yau T., Ross P., Mazzaferro V., Blanc J.-F., Ma Y.T., Yen C.J., Kocsis J. (2018). Phase II Studies with Refametinib or Refametinib plus Sorafenib in Patients with *RAS* -Mutated Hepatocellular Carcinoma. Clin. Cancer Res..

[55] Nault J.-C., Zucman-Rossi J. (2011). Genetics of Hepatobiliary Carcinogenesis. Semin Liver Dis..

[56] Boyault S., Rickman D.S., de Reyniès A., Balabaud C., Rebouissou S., Jeannot E., Hérault A., Saric J., Belghiti J., Franco D. (2007). Transcriptome Classification of HCC Is Related to Gene Alterations and to New Therapeutic Targets. Hepatology.

[57] Calderaro J., Couchy G., Imbeaud S., Amaddeo G., Letouzé E., Blanc J.-F., Laurent C., Hajji Y., Azoulay D., Bioulac-Sage P. (2017). Histological Subtypes of Hepatocellular Carcinoma Are Related to Gene Mutations and Molecular Tumour Classification. J. Hepatol..

[58] Hoshida Y., Nijman S.M.B., Kobayashi M., Chan J.A., Brunet J.-P., Chiang D.Y., Villanueva A., Newell P., Ikeda K., Hashimoto M. (2009). Integrative Transcriptome Analysis Reveals Common Molecular Subclasses of Human Hepatocellular Carcinoma. Cancer Res..

[59] Hirsch T.Z., Negulescu A., Gupta B., Caruso S., Noblet B., Couchy G., Bayard Q., Meunier L., Morcrette G., Scoazec J.-Y. (2020). BAP1 Mutations Define a Homogeneous Subgroup of Hepatocellular Carcinoma with Fibrolamellar-like Features and Activated PKA. J. Hepatol..

[60] Ziol M., Poté N., Amaddeo G., Laurent A., Nault J.-C., Oberti F., Costentin C., Michalak S., Bouattour M., Francoz C. (2018). Macrotrabecular-Massive Hepatocellular Carcinoma: A Distinctive Histological Subtype with Clinical Relevance. Hepatology.

[61] Montironi C., Castet F., Haber P.K., Pinyol R., Torres-Martin M., Torrens L., Mesropian A., Wang H., Puigvehi M., Maeda M. (2023). Inflamed and Non-Inflamed Classes of HCC: A Revised Immunogenomic Classification. Gut.

[62] Nowell P.C. (1976). The Clonal Evolution of Tumor Cell Populations: Acquired Genetic Lability Permits Stepwise Selection of Variant Sublines and Underlies Tumor Progression. Science.

[63] Lin D.-C., Mayakonda A., Dinh H.Q., Huang P., Lin L., Liu X., Ding L., Wang J., Berman B.P., Song E.-W. (2017). Genomic and Epigenomic Heterogeneity of Hepatocellular Carcinoma. Cancer Res..

[64] Guo L., Yi X., Chen L., Zhang T., Guo H., Chen Z., Cheng J., Cao Q., Liu H., Hou C. (2022). Single-Cell DNA Sequencing Reveals Punctuated and Gradual Clonal Evolution in Hepatocellular Carcinoma. Gastroenterology.

[65] Sun Y.-F., Wu L., Liu S.-P., Jiang M.-M., Hu B., Zhou K.-Q., Guo W., Xu Y., Zhong Y., Zhou X.-R. (2021). Dissecting Spatial Heterogeneity and the Immune-Evasion Mechanism of CTCs by Single-Cell RNA-Seq in Hepatocellular Carcinoma. Nat. Commun..

[66] Ho D.W.-H., Tsui Y.-M., Chan L.-K., Sze K.M.-F., Zhang X., Cheu J.W.-S., Chiu Y.-T., Lee J.M.-F., Chan A.C.-Y., Cheung E.T.-Y. (2021). Single-Cell RNA Sequencing Shows the Immunosuppressive Landscape and Tumor Heterogeneity of HBV-Associated Hepatocellular Carcinoma. Nat. Commun..

[67] Heinrich S., Craig A.J., Ma L., Heinrich B., Greten T.F., Wang X.W. (2021). Understanding Tumour Cell Heterogeneity and Its Implication for Immunotherapy in Liver Cancer Using Single-Cell Analysis. J. Hepatol..

[68] Zhai W., Lim T.K.-H., Zhang T., Phang S.-T., Tiang Z., Guan P., Ng M.-H., Lim J.Q., Yao F., Li Z. (2017). The Spatial Organization of Intra-Tumour Heterogeneity and Evolutionary Trajectories of Metastases in Hepatocellular Carcinoma. Nat. Commun..

[69] Friemel J., Rechsteiner M., Frick L., Böhm F., Struckmann K., Egger M., Moch H., Heikenwalder M., Weber A. (2015). Intratumor Heterogeneity in Hepatocellular Carcinoma. Clin. Cancer Res..

[70] Furuta M., Ueno M., Fujimoto A., Hayami S., Yasukawa S., Kojima F., Arihiro K., Kawakami Y., Wardell C.P., Shiraishi Y. (2017). Whole Genome Sequencing Discriminates Hepatocellular Carcinoma with Intrahepatic Metastasis from Multi-Centric Tumors. J. Hepatol..

[71] Tran N.H., Kisiel J., Roberts L.R. (2021). Using Cell-Free DNA for HCC Surveillance and Prognosis. JHEP Rep..

[72] Crowley E., Di Nicolantonio F., Loupakis F., Bardelli A. (2013). Liquid Biopsy: Monitoring Cancer-Genetics in the Blood. Nat. Rev. Clin Oncol..

[73] Diehl F., Schmidt K., Choti M.A., Romans K., Goodman S., Li M., Thornton K., Agrawal N., Sokoll L., Szabo S.A. (2008). Circulating Mutant DNA to Assess Tumor Dynamics. Nat. Med..

[74] Krebs M.G., Malapelle U., André F., Paz-Ares L., Schuler M., Thomas D.M., Vainer G., Yoshino T., Rolfo C. (2022). Practical Considerations for the Use of Circulating Tumor DNA in the Treatment of Patients With Cancer: A Narrative Review. JAMA Oncol..

[75] Rolfo C., Mack P., Scagliotti G.V., Aggarwal C., Arcila M.E., Barlesi F., Bivona T., Diehn M., Dive C., Dziadziuszko R. (2021). Liquid Biopsy for Advanced NSCLC: A Consensus Statement From the International Association for the Study of Lung Cancer. J. Thorac. Oncol..

[76] Gradishar W.J., Anderson B.O., Abraham J., Aft R., Agnese D., Allison K.H., Blair S.L., Burstein H.J., Dang C., Elias A.D. (2022). Breast Cancer, Version 3.2022, NCCN Clinical Practice Guidelines in Oncology. J. Natl Compr Canc Netw..

[77] Iizuka N., Sakaida I., Moribe T., Fujita N., Miura T., Stark M., Tamatsukuri S., Ishitsuka H., Uchida K., Terai S. (2006). Elevated Levels of Circulating Cell-Free DNA in the Blood of Patients with Hepatitis C Virus-Associated Hepatocellular Carcinoma. Anticancer. Res..

[78] Yan L., Chen Y., Zhou J., Zhao H., Zhang H., Wang G. (2018). Diagnostic Value of Circulating Cell-Free DNA Levels for Hepatocellular Carcinoma. Int. J. Infect. Dis..

[79] Qu C., Wang Y., Wang P., Chen K., Wang M., Zeng H., Lu J., Song Q., Diplas B.H., Tan D. (2019). Detection of Early-Stage Hepatocellular Carcinoma in Asymptomatic HBsAg-Seropositive Individuals by Liquid Biopsy. Proc. Natl. Acad. Sci. USA.

[80] Tao K., Bian Z., Zhang Q., Guo X., Yin C., Wang Y., Zhou K., Wan S., Shi M., Bao D. (2020). Machine Learning-Based Genome-Wide Interrogation of Somatic Copy Number Aberrations in Circulating Tumor DNA for Early Detection of Hepatocellular Carcinoma. EBioMedicine.

[81] Kisiel J.B., Dukek B.A., Kanipakam R.V.S.R., Ghoz H.M., Yab T.C., Berger C.K., Taylor W.R., Foote P.H., Giama N.H., Onyirioha K. (2019). Hepatocellular Carcinoma Detection by Plasma Methylated DNA: Discovery, Phase I Pilot, and Phase II Clinical Validation. Hepatology.

[82] Xu R., Wei W., Krawczyk M., Wang W., Luo H., Flagg K., Yi S., Shi W., Quan Q., Li K. (2017). Circulating Tumour DNA Methylation Markers for Diagnosis and Prognosis of Hepatocellular Carcinoma. Nat. Mater..

[83] Chalasani N.P., Ramasubramanian T.S., Bhattacharya A., Olson M.C., Edwards V.D.K., Roberts L.R., Kisiel J.B., Reddy K.R., Lidgard G.P., Johnson S.C. (2021). A Novel Blood-Based Panel of Methylated DNA and Protein Markers for Detection of Early-Stage Hepatocellular Carcinoma. Clin. Gastroenterol. Hepatol..

[84] Oussalah A., Rischer S., Bensenane M., Conroy G., Filhine-Tresarrieu P., Debard R., Forest-Tramoy D., Josse T., Reinicke D., Garcia M. (2018). Plasma MSEPT9: A Novel Circulating Cell-Free DNA-Based Epigenetic Biomarker to Diagnose Hepatocellular Carcinoma. EBioMedicine.

[85] Cai J., Chen L., Zhang Z., Zhang X., Lu X., Liu W., Shi G., Ge Y., Gao P., Yang Y. (2019). Genome-Wide Mapping of 5-Hydroxymethylcytosines in Circulating Cell-Free DNA as a Non-Invasive Approach for Early Detection of Hepatocellular Carcinoma. Gut.

[86] Kotoh Y., Suehiro Y., Saeki I., Hoshida T., Maeda M., Iwamoto T., Matsumoto T., Hidaka I., Ishikawa T., Takami T. (2020). Novel Liquid Biopsy Test Based on a Sensitive Methylated *SEPT9* Assay for Diagnosing Hepatocellular Carcinoma. Hepatol. Commun..

[87] Lin N., Lin Y., Xu J., Liu D., Li D., Meng H., Gallant M.A., Kubota N., Roy D., Li J.S. (2022). A Multi-analyte Cell-free DNA –Based Blood Test for Early Detection of Hepatocellular Carcinoma. Hepatol. Commun..

[88] Tokuhisa Y., Iizuka N., Sakaida I., Moribe T., Fujita N., Miura T., Tamatsukuri S., Ishitsuka H., Uchida K., Terai S. (2007). Circulating Cell-Free DNA as a Predictive Marker for Distant Metastasis of Hepatitis C Virus-Related Hepatocellular Carcinoma. Br. J. Cancer.

[89] Cai Z., Chen G., Zeng Y., Dong X., Li Z., Huang Y., Xin F., Qiu L., Xu H., Zhang W. (2019). Comprehensive Liquid Profiling of Circulating Tumor DNA and Protein Biomarkers in Long-Term Follow-Up Patients with Hepatocellular Carcinoma. Clin. Cancer Res..

[90] García-Fernández N., Macher H.C., Rubio A., Jiménez-Arriscado P., Bernal-Bellido C., Bellido-Díaz M.L., Suárez-Artacho G., Guerrero J.M., Gómez-Bravo M.A., Molinero P., Gahan P.B., Fleischhacker M., Schmidt B. (2016). Detection of P53 Mutations in Circulating DNA of Transplanted Hepatocellular Carcinoma Patients as a Biomarker of Tumor Recurrence. Circulating Nucleic Acids in Serum and Plasma—CNAPS IX..

[91] Zhu G., Liu W., Tang Z., Qu W., Fang Y., Jiang X., Song S., Wang H., Tao C., Zhou P. (2022). Serial Circulating Tumor DNA to Predict Early Recurrence in Patients with Hepatocellular Carcinoma: A Prospective Study. Mol. Oncol..

[92] Li C., Ho M., Lin Y., Tzeng S., Chen Y., Pai H., Wang Y., Chen C., Lee Y., Chen D. (2020). Cell-Free Virus-Host Chimera DNA From Hepatitis B Virus Integration Sites as a Circulating Biomarker of Hepatocellular Cancer. Hepatology.

[93] Ako S., Nouso K., Kinugasa H., Matsushita H., Terasawa H., Adachi T., Wada N., Takeuchi Y., Mandai M., Onishi H. (2020). Human Telomerase Reverse Transcriptase Gene Promoter Mutation in Serum of Patients with Hepatocellular Carcinoma. Oncology.

[94] Oh C.R., Kong S.-Y., Im H.-S., Kim H.J., Kim M.K., Yoon K.-A., Cho E.-H., Jang J.-H., Lee J., Kang J. (2019). Genome-Wide Copy Number Alteration and VEGFA Amplification of Circulating Cell-Free DNA as a Biomarker in Advanced Hepatocellular Carcinoma Patients Treated with Sorafenib. BMC Cancer.

[95] Hirai M., Kinugasa H., Nouso K., Yamamoto S., Terasawa H., Onishi Y., Oyama A., Adachi T., Wada N., Sakata M. (2021). Prediction of the Prognosis of Advanced Hepatocellular Carcinoma by *TERT* Promoter Mutations in Circulating Tumor DNA. J. Gastroenterol. Hepatol..

[96] Matsumae T., Kodama T., Myojin Y., Maesaka K., Sakamori R., Takuwa A., Oku K., Motooka D., Sawai Y., Oshita M. (2022). Circulating Cell-Free DNA Profiling Predicts the Therapeutic Outcome in Advanced Hepatocellular Carcinoma Patients Treated with Combination Immunotherapy. Cancers.

[97] Fujii Y., Ono A., Hayes C.N., Aikata H., Yamauchi M., Uchikawa S., Kodama K., Teraoka Y., Fujino H., Nakahara T. (2021). Identification and Monitoring of Mutations in Circulating Cell-Free Tumor DNA in Hepatocellular Carcinoma Treated with Lenvatinib. J. Exp. Clin. Cancer Res..

[98] von Felden J., Craig A.J., Garcia-Lezana T., Labgaa I., Haber P.K., D’Avola D., Asgharpour A., Dieterich D., Bonaccorso A., Torres-Martin M. (2021). Mutations in Circulating Tumor DNA Predict Primary Resistance to Systemic Therapies in Advanced Hepatocellular Carcinoma. Oncogene.

[99] Nakatsuka T., Nakagawa H., Hayata Y., Wake T., Yamada T., Nishibatake Kinoshita M., Nakagomi R., Sato M., Minami T., Uchino K. (2021). Post-Treatment Cell-Free DNA as a Predictive Biomarker in Molecular-Targeted Therapy of Hepatocellular Carcinoma. J. Gastroenterol..

[100] Alunni-Fabbroni M., Rönsch K., Huber T., Cyran C.C., Seidensticker M., Mayerle J., Pech M., Basu B., Verslype C., Benckert J. (2019). Circulating DNA as Prognostic Biomarker in Patients with Advanced Hepatocellular Carcinoma: A Translational Exploratory Study from the SORAMIC Trial. J. Transl. Med..

[101] Ikeda S., Lim J.S., Kurzrock R. (2018). Analysis of Tissue and Circulating Tumor DNA by Next-Generation Sequencing of Hepatocellular Carcinoma: Implications for Targeted Therapeutics. Mol. Cancer Ther..

[102] Cohen J.D., Li L., Wang Y., Thoburn C., Afsari B., Danilova L., Douville C., Javed A.A., Wong F., Mattox A. (2018). Detection and Localization of Surgically Resectable Cancers with a Multi-Analyte Blood Test. Science.

[103] Liu M.C., Oxnard G.R., Klein E.A., Swanton C., Seiden M.V., Liu M.C., Oxnard G.R., Klein E.A., Smith D., Richards D. (2020). Sensitive and Specific Multi-Cancer Detection and Localization Using Methylation Signatures in Cell-Free DNA. Ann. Oncol..

[104] Cavallone L., Aguilar-Mahecha A., Lafleur J., Brousse S., Aldamry M., Roseshter T., Lan C., Alirezaie N., Bareke E., Majewski J. (2020). Prognostic and Predictive Value of Circulating Tumor DNA during Neoadjuvant Chemotherapy for Triple Negative Breast Cancer. Sci. Rep..

[105] Tie J., Cohen J.D., Lahouel K., Lo S.N., Wang Y., Kosmider S., Wong R., Shapiro J., Lee M., Harris S. (2022). Circulating Tumor DNA Analysis Guiding Adjuvant Therapy in Stage II Colon Cancer. N. Engl. J. Med..

[106] Henriksen T.V., Tarazona N., Frydendahl A., Reinert T., Gimeno-Valiente F., Carbonell-Asins J.A., Sharma S., Renner D., Hafez D., Roda D. (2022). Circulating Tumor DNA in Stage III Colorectal Cancer, beyond Minimal Residual Disease Detection, toward Assessment of Adjuvant Therapy Efficacy and Clinical Behavior of Recurrences. Clin. Cancer Res..

[107] Chaudhuri A.A., Chabon J.J., Lovejoy A.F., Newman A.M., Stehr H., Azad T.D., Khodadoust M.S., Esfahani M.S., Liu C.L., Zhou L. (2017). Early Detection of Molecular Residual Disease in Localized Lung Cancer by Circulating Tumor DNA Profiling. Cancer Discov..

[108] Garcia-Murillas I., Schiavon G., Weigelt B., Ng C., Hrebien S., Cutts R.J., Cheang M., Osin P., Nerurkar A., Kozarewa I. (2015). Mutation Tracking in Circulating Tumor DNA Predicts Relapse in Early Breast Cancer. Sci. Transl. Med..

[109] Tie J., Cohen J.D., Wang Y., Li L., Christie M., Simons K., Elsaleh H., Kosmider S., Wong R., Yip D. (2019). Serial Circulating Tumour DNA Analysis during Multimodality Treatment of Locally Advanced Rectal Cancer: A Prospective Biomarker Study. Gut.

[110] Yang J., Gong Y., Lam V.K., Shi Y., Guan Y., Zhang Y., Ji L., Chen Y., Zhao Y., Qian F. (2020). Deep Sequencing of Circulating Tumor DNA Detects Molecular Residual Disease and Predicts Recurrence in Gastric Cancer. Cell Death Dis..

[111] Leighl N.B., Page R.D., Raymond V.M., Daniel D.B., Divers S.G., Reckamp K.L., Villalona-Calero M.A., Dix D., Odegaard J.I., Lanman R.B. (2019). Clinical Utility of Comprehensive Cell-Free DNA Analysis to Identify Genomic Biomarkers in Patients with Newly Diagnosed Metastatic Non–Small Cell Lung Cancer. Clin. Cancer Res..

[112] Kato S., Schwaederlé M.C., Fanta P.T., Okamura R., Leichman L., Lippman S.M., Lanman R.B., Raymond V.M., Talasaz A., Kurzrock R. (2019). Genomic Assessment of Blood-Derived Circulating Tumor DNA in Patients With Colorectal Cancers: Correlation With Tissue Sequencing, Therapeutic Response, and Survival. JCO Precis. Oncol..

[113] Garlan F., Laurent-Puig P., Sefrioui D., Siauve N., Didelot A., Sarafan-Vasseur N., Michel P., Perkins G., Mulot C., Blons H. (2017). Early Evaluation of Circulating Tumor DNA as Marker of Therapeutic Efficacy in Metastatic Colorectal Cancer Patients (PLACOL Study). Clin. Cancer Res..

[114] Zhu A.X., Kang Y.-K., Yen C.-J., Finn R.S., Galle P.R., Llovet J.M., Assenat E., Brandi G., Pracht M., Lim H.Y. (2019). Ramucirumab after Sorafenib in Patients with Advanced Hepatocellular Carcinoma and Increased α-Fetoprotein Concentrations (REACH-2): A Randomised, Double-Blind, Placebo-Controlled, Phase 3 Trial. Lancet Oncol..

[115] Diaz Jr L.A., Williams R.T., Wu J., Kinde I., Hecht J.R., Berlin J., Allen B., Bozic I., Reiter J.G., Nowak M.A. (2012). The Molecular Evolution of Acquired Resistance to Targeted EGFR Blockade in Colorectal Cancers. Nature.

[116] Misale S., Yaeger R., Hobor S., Scala E., Janakiraman M., Liska D., Valtorta E., Schiavo R., Buscarino M., Siravegna G. (2012). Emergence of KRAS Mutations and Acquired Resistance to Anti-EGFR Therapy in Colorectal Cancer. Nature.

[117] Zheng D., Ye X., Zhang M.Z., Sun Y., Wang J.Y., Ni J., Zhang H.P., Zhang L., Luo J., Zhang J. (2016). Plasma EGFR T790M CtDNA Status Is Associated with Clinical Outcome in Advanced NSCLC Patients with Acquired EGFR-TKI Resistance. Sci. Rep..

[118] Fribbens C., Garcia Murillas I., Beaney M., Hrebien S., O’Leary B., Kilburn L., Howarth K., Epstein M., Green E., Rosenfeld N. (2018). Tracking Evolution of Aromatase Inhibitor Resistance with Circulating Tumour DNA Analysis in Metastatic Breast Cancer. Ann. Oncol..

[119] Sartore-Bianchi A., Pietrantonio F., Lonardi S., Mussolin B., Rua F., Crisafulli G., Bartolini A., Fenocchio E., Amatu A., Manca P. (2022). Circulating Tumor DNA to Guide Rechallenge with Panitumumab in Metastatic Colorectal Cancer: The Phase 2 CHRONOS Trial. Nat. Med..

